# The Atherosclerotic Plaque Microenvironment as a Therapeutic Target

**DOI:** 10.1007/s11883-025-01294-y

**Published:** 2025-04-02

**Authors:** Rajan Pandit, Arif Yurdagul

**Affiliations:** 1 Department of Molecular and Cellular Physiology, LSU Health Sciences Center at Shreveport, Shreveport, LA USA; 2 Department of Pathology and Translational Pathobiology, LSU Health Sciences Center at Shreveport, Shreveport, LA USA

**Keywords:** Atherosclerosis, Atherosclerotic plaque microenvironment, Cardiovascular disease, Extracellular matrix remodeling, Inflammation resolution

## Abstract

**Purpose of Review:**

Atherosclerosis is traditionally viewed as a disease triggered by lipid accumulation, but growing evidence underscores the crucial role of the plaque microenvironment in disease progression. This review explores recent advances in understanding how cellular and extracellular components of the plaque milieu drive atherosclerosis, with a focus on leveraging these microenvironmental factors for therapeutic intervention. This review highlights recent advances in cell-cell crosstalk and matrix remodeling, offering insights into innovative therapeutic strategies for atherosclerotic cardiovascular disease.

**Recent Findings:**

While atherosclerosis begins with the subendothelial retention of apolipoprotein B (ApoB)-containing lipoproteins​, its progression is increasingly recognized as a consequence of complex cellular and extracellular dynamics within the plaque microenvironment. Soluble factors and extracellular matrix proteins shape mechanical properties and the biochemical landscape, directly influencing cell behavior and inflammatory signaling. For instance, the deposition of transitional matrix proteins, such as fibronectin, in regions of disturbed flow primes endothelial cells for inflammation​. Likewise, impaired clearance of dead cells and chronic extracellular matrix remodeling contribute to lesion expansion and instability, further exacerbating disease severity.

**Summary:**

Targeting the plaque microenvironment presents a promising avenue for stabilizing atherosclerotic lesions. Approaches that enhance beneficial cellular interactions, such as boosting macrophage efferocytosis to resolve inflammation while mitigating proatherogenic signals like integrin-mediated endothelial activation, may promote fibrous cap formation and reduce plaque vulnerability. Harnessing these mechanisms may lead to novel therapeutic approaches aimed at modifying the plaque microenvironment to combat atherosclerotic cardiovascular disease.

## Introduction

### Atherosclerosis Initiation and Progression

Atherosclerosis is a progressive inflammatory disease that begins with the subendothelial retention of cholesterol-rich ApoB-containing lipoproteins in medium- and large-sized arteries [1–3]. According to the response-to-retention model, this trapping of lipoproteins in the arterial intima represents the initial key event in atherosclerosis development [4, 5]. The retained low-density lipoprotein (LDL) particles become modified (e.g., by oxidation, glycation, or aggregation) and trigger endothelial cell activation and dysfunction. Endothelial dysfunction, characterized by increased permeability, reduced nitric oxide bioavailability, and upregulation in adhesion molecules, establishes a proinflammatory microenvironment that promotes immune cell infiltration [6–8]. Monocytes adhere to activated endothelium and transmigrate into the arterial wall, where they differentiate into macrophages and uptake lipids to form foam cells [9]. The persistent accumulation of lipids and proinflammatory mediators also impairs the clearance of dead cells by macrophages, a process termed ‘efferocytosis’, leading to post-apoptotic necrosis and necrotic core formation in plaques [10]. This sequence produces unstable atherosclerotic plaques with large necrotic cores and thin fibrous caps, which may either rupture or undergo superficial plaque erosion, increasing the risk of thromboembolic cardiovascular events, such as myocardial infarction and stroke [11–13].

Clinical trials targeting inflammation have confirmed the link between lesional inflammation and cardiovascular events. The Canakinumab Anti-Inflammatory Thrombosis Outcome Study (CANTOS) showed that IL-1β inhibition reduced recurrent events independent of lipid-lowering but was associated with a higher incidence of fatal infections [14, 15]. Similarly, the Colchicine Cardiovascular Outcomes Trial (COLCOT) demonstrated reduced ischemic events with low-dose colchicine, albeit with a slight increase in pneumonia in those receiving treatment [15]. These landmark trials underscore that while reducing inflammation can mitigate atherosclerosis, preserving host defenses remains essential. Thus, therapies that resolve inflammation without broadly suppressing immunity are a promising approach to combat atherosclerotic cardiovascular disease.

### The Atherosclerotic Plaque Microenvironment

The plaque microenvironment– comprising extracellular matrix (ECM) proteins, biomechanical forces, and soluble factors– plays a pivotal role in atherogenesis. In healthy arteries, the intimal ECM is primarily composed of basement membrane proteins such as collagen IV, laminin, perlecan, and nidogen, which provide structural support and maintain cellular homeostasis [16, 17]. In atherosclerosis, this ECM undergoes extensive remodeling [18]. One of the most significant changes is the deposition of transitional matrix proteins in the subendothelial space, replacing the normal basement membrane with a proinflammatory provisional matrix. For instance, fibronectin accumulates at atheroprone sites exposed to disturbed flow even before the appearance of plaque formation [19]. This fibronectin-rich matrix “primes” endothelial cells for inflammatory activation by stimuli such as oxidized LDL (oxLDL), in contrast to the atheroprotective signaling elicited by laminin and collagen IV in healthy arteries [2, 18, 20]. Beyond fibronectin, other matrix components also contribute to the lesional microenvironment. Negatively charged glycosaminoglycans (e.g. hyaluronan, heparan sulfate, chondroitin sulfate) within proteoglycans bind and sequester growth factors such as platelet-derived growth factor (PDGF), transforming growth factor β (TGFβ), hepatocyte growth factor (HGF), fibroblast growth factor (FGF), and insulin-like growth factor (IGF), modulating the proliferation and migration of cells [21–23]​. As atherosclerosis progresses, lesional cells alter the microenvironment by releasing cytokines, chemokines, and metabolites that propagate a self-amplifying cycle of chronic inflammation and fibrotic responses that drive more inflammation and ECM remodeling [23, 24]. However, important aspects of ECM remodeling in atherosclerosis remain poorly understood. The precise mechanisms governing the transition from a healthy basement membrane to a proatherogenic matrix, as well as how altered ECM composition sustains chronic inflammation and disease progression, still require further investigation. Because the plaque microenvironment plays a central role in regulating cell behavior, targeting this niche is therapeutically attractive. The following sections examine key pathways among lesional cells and discuss how modulating these interactions or the ECM could stabilize plaques.

### Endothelial-Leukocyte Crosstalk

#### Endothelial Cell Activation

Atherosclerotic lesions preferentially form at arterial branch points and curvatures, where disturbed blood flow creates low and oscillatory shear stress. In contrast, high, unidirectional laminar flow is atheroprotective by driving nuclear localization of KLF2 and KLF4, which in turn enhances endothelial nitric oxide synthase (eNOS) expression and nitric oxide production [25, 26]. Recent studies indicate that atheroprotective shear stress induces HEG1-mediated KLF2 and KLF4 expression, and endothelial HEG1 is downregulated in individuals with advanced atherosclerosis. In contrast, disturbed flow suppresses the atheroprotective transcription factors KLF2 and KLF4 and activates NF-κB and other inflammatory pathways in endothelial cells [27, 28]. Endothelial cells also secrete chemokines such as CCL2 (MCP-1), CCL5, and CX3CL1 that diffuse into the bloodstream. Once captured by endothelial selectins, circulating monocytes and other leukocytes firmly adhere through integrins, such as VLA-4 and LFA-1, which bind to endothelial VCAM-1 and ICAM-1. This multistep adhesion cascade (summarized in Table 1) guides leukocytes across the endothelium into the intima​. Once transmigrated, monocytes become activated by macrophage colony-stimulating factor (M-CSF), CCL2, and other factors that drive their differentiation into macrophages. The local microenvironment will then shape the macrophage phenotype, as will be discussed later.

**Table 1:** Major Leukocyte-Endothelial adhesion molecules [101–103]

Leukocytes Molecules/Receptors	Endothelial or ECM ligands	Role
L-selectin, VLA-4 (α_4_β_1_)	s-Le^x^, VCAM-1 or FN	tethering
L-selectin, VLA-4, ESL-1, PSGL-1	s-Le^x^, VCAM-1, E-selectin, P-selectin	rolling
Chemokine-R, PAF-R	Chemokines, PAF	activation
LFA-1(α_L_β2); Mac − 1 (αMβ2); VLA-4 (α_4_β_1_)	ICAM-1,2,3 and JAM-A; ICAM1,2, FN, VN; VCAM-1, FN	adhesion
Mac-1 (LFA-1)	ICAM-1 (ICAM-2)	migration
PECAM-1, CD99	PECAM-1 (CD31), CD99	transmigration
α_6_β_1_	Laminin	migrating through basal lamina
Mac-1, α_5_β_1_	FG, FN	interact with fibronectin
JAM A, B	JAM A, B, C	homophilic/heterophilic interaction for firm interaction

ESL-1, E-selectin ligand 1; s-Le^x^ sialyl-Lewis x antigen; VCAM-1, vascular cell adhesion molecule 1; PSGL-1, P-selectin glycoprotein ligand 1; LFA-1, lymphocyte function-associated antigen 1; Mac-1, macrophage-1 antigen; PAF, platelet-activating factor; PAF-R, PAF receptor; ICAM, intercellular adhesion molecule; PECAM-1, platelet/endothelial cell adhesion molecule 1; PECAM-1, a6b1, VLA-6, the integrin responsible for binding laminin. FN, fibronectin; FG, fibrinogen; VN, vitronectin

#### Response to Oxidized LDL

**Fig. 1:**
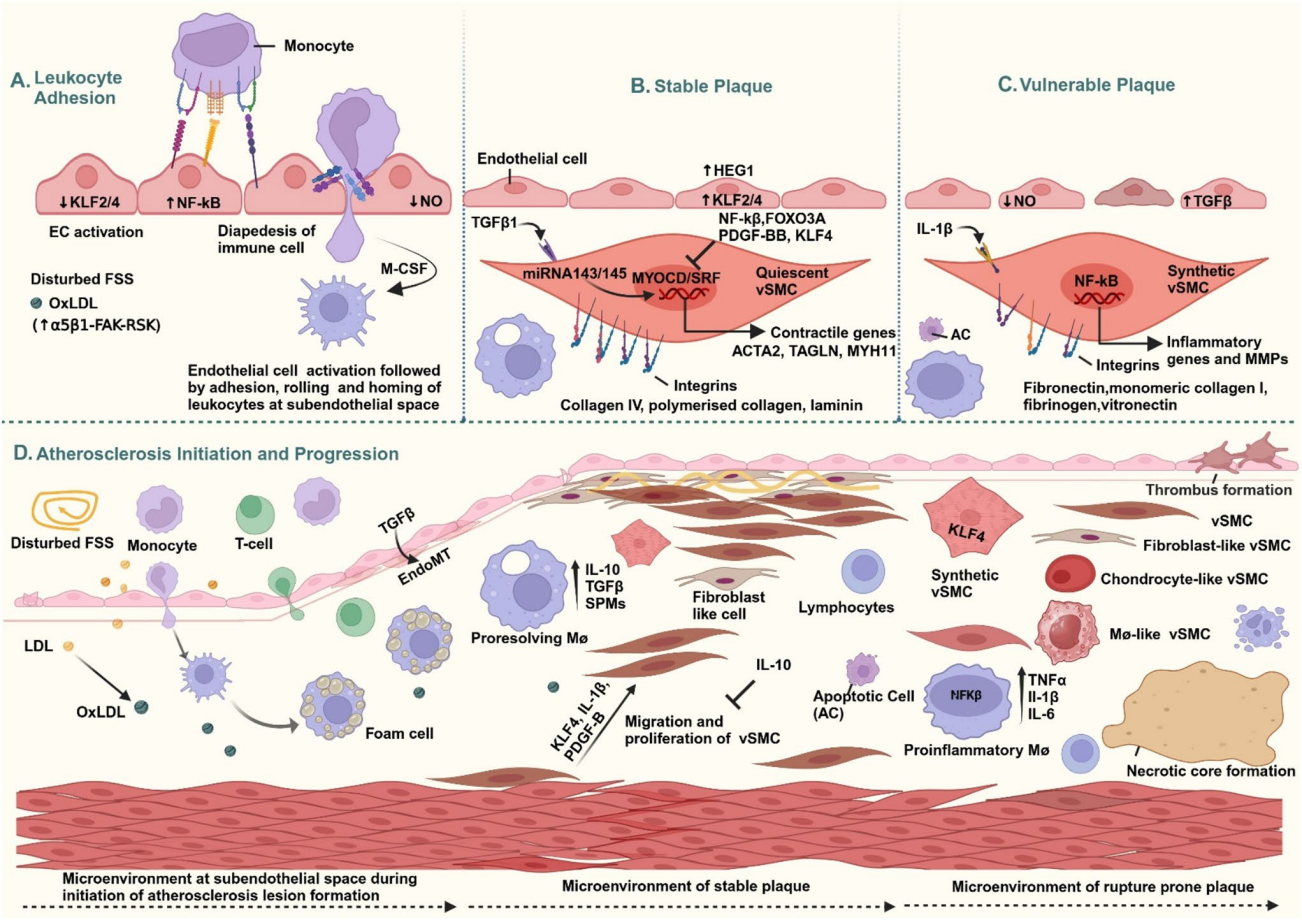
Atherosclerosis Initiation and Progression

Positively charged ApoB-containing lipoproteins traverse the endothelium via paracellular or transcytosis routes [8]. Once in the subendothelial space, LDL undergoes oxidative modification and becomes trapped within the negatively charged, proteoglycan-rich subendothelial matrix [8, 29–31]. These modified LDLs disrupt endothelial cell function by activating NF-κB, increasing permeability, reducing nitric oxide production, upregulating adhesion molecules, and enhancing leukocyte infiltration (Fig. 1A) [32, 33].

#### Endothelial Responses Modulated by the ECM

The composition of the subendothelial ECM not only regulates LDL retention but also provides signals to endothelial cells that modulate inflammation [34, 35]. Endothelial cells interact with the basement membrane proteins via the integrin family of cell-matrix receptors. Such interactions can transmit atheroprotective signals. As one example, endothelial interactions with basement membrane proteins via integrin α2β1 signaling blunt disturbed flow and oxLDL-induced inflammation [7, 24]. However, in early atherosclerosis, as the basement membrane is being replaced by fibronectin, endothelial cells switch to using integrin α5β1 for adhesion. Activation of integrin α5β1 by fibronectin amplifies endothelial cell activation, creating a positive feedback loop in disturbed flow regions [35, 36]. Thus, the arterial microenvironment, from biomechanical forces to matrix composition, critically regulates endothelial behavior and, consequently, leukocyte recruitment in atherosclerosis.

## Endothelial-Vascular Smooth Muscle Cell Crosstalk

Endothelial cells and vascular smooth muscle cells (vSMCs) are in close proximity in the arterial microenvironment and communicate through various mechanisms, including direct cell-cell contact, paracrine signaling, and extracellular vesicles [37]. In a healthy vessel, this endothelial–vSMC crosstalk regulates vascular tone and structure. For instance, the vasodilatory signal nitric oxide is released by endothelial cells that act on adjacent vSMCs to induce relaxation, thus maintaining appropriate vessel caliber and blood pressure. Under laminar flow conditions, endothelial production of nitric oxide is high and vSMCs remain in a contractile, quiescent state. Disturbed flow, however, not only activates endothelial cells (as described above) but also alters the signals sent to vSMCs. ECs exposed to low/oscillatory shear can exhibit an EndMT (endothelial-to-mesenchymal transition) phenotype, producing factors like TGFβ2 and IL-1β that impact vSMCs​. TGFβ2 from activated endothelial cells can drive vSMCs toward a more synthetic or matrix-producing vSMC phenotype (Fig. 1B)​. Alternatively, endothelial-derived IL-1β has been shown to stimulate vSMC migration and proliferation [38, 39]. These effects illustrate how inflammatory crosstalk from the endothelium can drive vSMCs away from their contractile phenotype.

Another key mode of communication between endothelial cells and vSMCs involves microRNAs (miRNAs), which can be transferred directly or via extracellular vesicles. A notable example is the transfer of miR-143/145 from vSMCs to endothelial cells. These miRNAs, highly expressed in vSMCs, promote the contractile SMC phenotype. When endothelial cells produce TGFβ during direct interactions with vSMCs, it induces the formation of membrane nanotubes that facilitate the transfer of miR-143/145 from vSMCs to endothelial cells. The uptake of these miRNAs suppresses endothelial angiogenic activity and stabilizes vessels [40]​. This exchange creates a protective feedback loop, with vSMCs helping to establish an atheroprotective, quiescent endothelial state. However, endothelial-vSMC crosstalk can become harmful in disease settings. Disturbed flow and chronic inflammation can reduce the transfer of beneficial signals, such as miR-143/145 while amplifying detrimental ones. For instance, activated endothelial cells may release factors or vesicles that drive vSMC dedifferentiation, promoting a less stable phenotype. Simultaneously, inflamed vSMCs may secrete proteases or cytokines that further damage the endothelium, exacerbating vascular dysfunction [41]. Because of this dynamic interplay, strategies that disrupt maladaptive endothelial-vSMC crosstalk or restore its protective functions represent a promising therapeutic strategy to attenuate atherosclerosis.

## Vascular Smooth Muscle Cell Phenotypic Modulation in the Plaque Microenvironment

Vascular SMCs are traditionally regarded as the structural cells of the arterial wall, responsible for both vasoconstriction and dilation [42]. Under normal conditions, vSMCs exhibit a contractile phenotype, characterized by a high propensity to constrict when necessary and a low rate of proliferation and migration. These vSMCs express high levels of contractility-related genes, including alpha smooth muscle actin (*ACTA2*), calponin (*CNN1*), transgelin (*TAGLN*), smoothelin (*SMTN*), and SM-MHC (*MYH11*) (Fig. 1B). This contractile phenotype is primarily maintained by myocardin, which interacts with serum response factor (SRF) to drive the expression of contractile genes [43–46].

In atherosclerosis, vSMCs are exposed to inflammatory cytokines, growth factors, and lipid mediators that can trigger phenotypic modulation, a process in which contractile markers are downregulated and acquire features associated with other cell types. Drivers of this switch include KLF4, NF-kB, and forkhead box O3a (FOXO3A), which become activated in vSMCs by atherogenic stimuli and inhibit myocardin function. This results in a loss in the contractile phenotype and the emergence of a synthetic, migratory, and proliferative phenotype (Fig. 1B). Phenotypically modulated vSMCs carry the potential to transdifferentiate into cells resembling macrophages, mesenchymal stem cells, osteochondrogenic cells, or fibroblasts, and single-cell RNA sequencing coupled with lineage-tracing studies have revealed that a significant fraction of cells within advanced plaques that appear “macrophage-like” or “foam cell-like” originate from vSMCs rather than monocyte-derived macrophages [47, 48]. These vSMC-derived foam cells often have impaired cholesterol efflux capacity relative to *bona fide* macrophages, which may exacerbate lipid accumulation in atherosclerosis plaques.

Smooth muscle cells interact with ECM proteins via the integrin family of cell-matrix receptors, and integrin-mediated signaling is crucial in determining vSMC phenotype and alters atherosclerosis progression (Fig. 1B and C). For instance, disrupting vSMC interactions with matrix proteins, particularly fibronectin, using α5β1 or αvβ3 inhibitors has been shown to reduce atherosclerotic plaque size. Notably, integrin αvβ3 inhibition decreases fibrous cap formation [7, 35]. Interestingly, while depleting fibronectin blocks endothelial cell activation, it simultaneously inhibits migration and proliferation of vSMCs, resulting in thin fibrous caps and poorly assembled ECM networks [19]. Vascular SMCs serve as a primary source of collagen within the lesional microenvironment, reinforcing the fibrous cap and enhancing plaque stability. However, in inflamed atheromas, macrophage-derived proteases, particularly MMPs, actively degrade collagen, weaken the protective fibrous cap, and compromise plaque stability, increasing the risk of plaque rupture [49, 50]. However, phenotypic switching of vSMCs is double-edged, whereby the synthetic state contributes to plaque expansion and instability by secreting inflammatory cytokines and the matrix-degrading enzymes matrix metalloproteases (MMPS) but can also produce ECM proteins that form the protective fibrous cap, an essential feature for stability. Interestingly, fibronectin in the lesional microenvironment promotes phenotypic switching that initially participates in fibrous cap formation, whereas a collagen-rich matrix can push vSMCs toward a more contractile, matrix-producing phenotype that stabilizes the cap. This dynamic underscores the complex role of vSMCs and highlights the importance of understanding the tissue microenvironment in atherosclerosis.

Indeed, approaches that return synthetic vSMCs towards a contractile phenotype can stabilize plaques. For example, microRNA-143/145, which promotes the contractile program in vSMCs, is protective, and deleting these miRNAs accelerates atherosclerosis. Similarly, TGFβ1 signaling sustains a differentiated state and increases collagen production [51, 52]. In contrast, KLF4 is a key driver of vSMC dedifferentiation during atherosclerosis (Fig. 1B). Conditional deletion of KLF4 in vSMCs of atheroprone mice fed a high-fat diet reduces lesion size, increases fibrous cap thickness, and decreases vSMC-derived macrophages and mesenchymal stem-like cells while increasing αSMA^+^ cells [53, 54]. Advancements in single-cell RNA sequencing (scRNA-seq) and lineage-tracing methods have uncovered distinct vSMC phenotypes during atherogenesis [55]. Recent findings have revealed at least four distinct phenotypes based on marker expression: contractile-like, fibroblast-like, chondrocyte-like, and macrophage-like. Further studies indicate that macrophages modulate vSMC function during atherosclerosis progression through IL-1β, whereby macrophage-derived IL-1β influences vSMC phenotype [55, 56]. Therapeutically targeting pathways such as KLF4 or NF-κB in vSMCs or bolstering pro-contractile signals (myocardin/SRF, TGFβ, miR-143/145) could tilt vSMCs toward a phenotype that stabilizes plaques rather than destabilizes them (Fig. 1B).

## Efficient Efferocytosis by Macrophages in Inflammation Resolution

Efferocytosis is the process by which macrophages bind, engulf, and digest apoptotic cells, ensuring the efficient clearance of cellular debris and maintaining tissue homeostasis. Proresolving macrophages play a key role in resolving inflammation and preventing post-apoptotic necrosis by efficiently clearing apoptotic cells. When macrophages consume apoptotic cells, they respond by suppressing proinflammatory cytokine production and releasing anti-inflammatory and proresolving mediators [57–59]. For instance, efferocytosis stimulates the secretion of specialized proresolving lipid mediators (SPMs) and anti-inflammatory cytokines such as IL-10 and TGFβ (Fig. 1D). IL-10 is particularly important for inflammation resolution, as it enhances cholesterol efflux via activating the PPARγ-LXR pathway. This upregulates the cholesterol transporters ABCA1 and ABCG1 while simultaneously reducing TNFα, IL-6, and MCP-1 [60–65]. One mechanism by which IL-10 inhibits inflammation is through induction of the transcriptional regulator Bcl-3, which interferes with NF-κB signaling to reduce IL-6 production in response to Toll-like receptor activation [66, 67]. Macrophages engulfing apoptotic cells also metabolize the contents of those cells. For example, the metabolism of apoptotic cell-derived arginine by macrophages leads to the production of polyamines (such as putrescine and spermidine) that further promote resolution, and spermidine and spermine can suppress inflammasome activation and IL-1β release [60, 68, 69]. Additionally, the breakdown of fatty acids and cholesterol from ingested apoptotic cells generates SPMs and oxysterols that engage nuclear receptors, such as LXR, to increase the expression of the key apoptotic cell receptor MerTK [63].

In advanced atherosclerotic lesions, macrophage efferocytosis becomes defective. As macrophages are overwhelmed by excess apoptotic cells and exposed to an inflamed microenvironment, their capacity to phagocytose and process dead cells diminishes [70]. Impaired efferocytosis leads to the accumulation of uncleared apoptotic bodies, which undergo post-apoptotic necrosis– releasing cytotoxic contents and proinflammatory molecules into the plaque​. The outcome is an expanding necrotic core and heightened inflammation, conditions strongly linked to plaque instability [71]​. Consistently, genetic and pharmacological approaches that restore efferocytosis enhance features associated with plaque stability, including smaller necrotic cores and increased fibrous cap thickness [72]. For example, blockade of the “don’t eat me” signal CD47, which normally inhibits macrophage phagocytosis via SIRPα, with antagonistic antibodies can restore efferocytosis, leading to enhanced clearance of apoptotic cells and reduced necrotic core size, and overall plaque stabilization [73]​. Similarly, overexpressing a cleavage-resistant MerTK or targeting negative regulators of MerTK, such as CAMKIIγ, improves features of plaque stability [74, 75].

A recently discovered feature of efferocytosis is that it can trigger a proliferative burst of macrophages with a proresolving phenotype. Apoptotic cell-derived nucleotides activate a DNA-PK–Akt–Myc pathway and drive the expansion of a macrophage subpopulation specialized in resolution​ through a process termed “efferocytosis-induced macrophage proliferation” (EIMP). These macrophages produce high levels of IL-10 and TGFβ and can rapidly engulf additional apoptotic cells, thus forming a positive feedback loop for restoring homeostasis [58]. However, this beneficial cycle is easily disrupted in an inflamed microenvironment. TNFα and IL-1β, which are abundant in advanced atherosclerosis, can promote ADAM17-dependent proteolytic cleavage of MerTK, substantially disabling efferocytosis​ [76]. Thus, restoring efferocytosis is an attractive therapeutic strategy to stabilize rupture-prone atheromas.

### Crosstalk between Macrophages and Vascular Smooth Muscle Cells

Interactions between macrophages and vSMCs within the plaque significantly influence whether a lesion progresses towards instability or heals into a stable fibrous plaque– exerting both beneficial and detrimental effects. On the beneficial side, macrophages engaged in efferocytosis release mediators that support vSMC function. For example, IL-10 attenuates PDGF- or LPS-induced vSMC proliferation and migration, thereby potentially limiting intimal hyperplasia [66]​ [77]. As previously mentioned, TGFβ promotes the contractile phenotype in vSMCs while also stimulating collagen synthesis​. It also suppresses vSMC expression of inflammatory mediators, such as inducible nitric oxide synthase (iNOS) and IL-6, further promoting a stable plaque environment [78–80]​. Additionally, macrophage-derived metabolites from efferocytosis serve as important signals for vSMCs. One notable example is lactate, whereby recent studies indicate that macrophage metabolizing apoptotic cells produce lactate, which in turn signals vSMCs to adopt a reparative, matrix-producing state [81]. Polyamines produced by macrophages following efferocytosis may have similar pro-fibrotic effects on vSMCs [23, 82]. Together, these interactions suggest that efferocytosis by macrophages promotes vSMC-mediated plaque stability​.

Conversely, proinflammatory macrophages can drive vSMC dysfunction, contributing to plaque instability. For instance, in co-culture experiments, oxLDL-activated macrophages have been shown to trigger a phenotypic switch in nearby vSMCs, pushing them toward a macrophage-like state through inflammasome signaling [83]. Additionally, macrophages release proteases, such as MMP-9 and MMP-13, which actively degrade collagen and elastin within the plaque microenvironment [84, 85]. Excessive macrophage activation can weaken the fibrous cap by driving collagen degradation faster than vSMCs can produce it, ultimately thinning the fibrous cap and increasing plaque instability​. Moreover, proinflammatory mediators, such as TNFα, can induce vSMC apoptosis while simultaneously suppressing collagen synthesis, further weakening the fibrous cap [86]​. Emerging evidence suggests that macrophages contribute to plaque calcification by secreting factors that stimulate an osteogenic program in vSMCs, potentially rendering plaques more susceptible to interfacial debonding [87] (Table 2).​ Ultimately, the impact of macrophage-SMC interactions on plaque stability hinges on the balance between resolving and inflammatory macrophage phenotypes.

**Table 2:** Molecular and cellular interactions between lesional cells

Soluble Factors Source (vSMCs/ Macrophages)	Signaling/Role	Ref
CCL2, CXCL1, BMPs from vSMCs	recruit macrophages (Mɸs)	[104–106]
TNFα from macrophages	atherosclerotic plaque calcification formation via BMP-2 expression in VSMCs	[87]
MMP1/9 from both cells	reduce collagen synthesis (col l/IIII, elastin) and degrade ECM of the fibrous cap	[107]
IL-1β, TLR-2, and VEGF-A from both cells	reduce plaque stability through inflammation, neovascularization, and calcification	[107]
IL-5 from macrophage	reduce inflammation and reduced Ang II-induced vSMC apoptosis within the aorta	[86]
oxLDL-activated monocytes co-cultured with vSMCs	nucleotide-binding oligomerization domain-like receptor protein 3 (NLRP3) inflammasome activation in VSMCs, transform vSMCs into a macrophage-like phenotype, gasdermin D-dependent pyroptosis of vSMCs	[83, 108]
Transfer of atherosclerotic factors via extracellular vesicles.	macrophages transfer miRNA, LDL, and protein cocktails to other macrophages and VSMCs.	[109, 110]

## Therapeutic Strategies Targeting the Plaque Microenvironment

Growing insights into the plaque microenvironment have driven the development of preclinical therapies that act directly on lesional cells rather than targeting systemic risk factors alone. One promising approach involves engineering nanoparticles to deliver drugs directly to atherosclerotic lesions, enhancing local bioavailability while minimizing off-target effects. Similarly, nanoparticles decorated with macrophage-targeting ligands have been used to deliver anti-inflammatory agents, such as IL-10, colchicine, and rapamycin, effectively reducing vascular inflammation [88–90]​. Others have delivered payloads to enhance efferocytosis, such as nanoparticles presenting MerTK on their surface, which promote apoptotic cell clearance and significantly attenuate atherosclerosis in mice [91]​. A multi-targeted strategy using RNAi delivered by nanoparticles to silence the adhesion molecules ICAM-1, VCAM-1, and E-selectin simultaneously reduces leukocyte recruitment and plaque inflammation [92].

Recognizing the role of lipid and metabolic signals in lesional cells, nuclear receptor agonists have garnered substantial attention. Encapsulating the LXR agonist GW3965 in targeted nanoparticles enhanced cholesterol removal from plaque macrophages while avoiding hepatic steatosis, resulting in overall anti-atherogenic benefits [93]. Similarly, activating PPARγ, which promotes IL-10 production and enhances efferocytosis, has been achieved using drugs such as pioglitazone, and pioglitazone-loaded nanoparticles were found to prevent features of plaque instability in mice by driving macrophage polarization towards a proresolving phenotype [94]. Notably, enhancing apoptotic cell clearance by macrophages has emerged as a promising strategy for plaque stability. One approach targets the inhibitory CD47-SIRPα pathway, where blocking the “don’t eat me” signal CD47 with antagonistic antibodies or peptides can reactivate macrophage efferocytosis, as demonstrated by reduced necrotic cores and smaller lesions in treated animals​. Another approach is to supplement pro-efferocytic factors [95]. For instance, injecting recombinant annexin A1 or resolvin D1 boosts the engulfment capacity of phagocytes [96]. Additionally, the novel strategy of delivering the MerTK receptor itself to lesions via nanocarriers fused with MerTK ectodomains has been used to directly promote apoptotic cell uptake in plaques ​ [91, 97].

Building on clinical trial successes, therapies that blunt specific cytokine signals in plaques are being refined. IL-1β neutralization with canakinumab has already been proven to reduce cardiovascular events [14]. In addition to lowering inflammation, IL-1β inhibition promotes the accumulation of fibrous cap–forming fibroblast-like cells originating from vSMCs and other stromal cells, which increases cap thickness [56]. This finding suggests IL-1β blockade not only reduces inflammation but also favorably alters cell composition in the plaque. Meanwhile, low-dose colchicine (which broadly dampens inflammation, including IL-1β and IL-18 production from macrophages) has emerged as a potential adjunct therapy for coronary disease [15]. Ongoing work aims to maximize the anti-inflammatory benefit of these agents while minimizing immunosuppressive risks.

An innovative area of research involves reprogramming immune cells to an atheroprotective state ex vivo before reintroduction. Monocytes can be metabolically “trained” to adopt a proresolving phenotype. For example, treating human monocytes with 4-phenylbutyric acid (4-PBA) ex vivo induces a sustained anti-inflammatory phenotype. When reintroduced into mice, these 4-PBA-trained monocytes home to lesions and ameliorate atherosclerosis​. Mechanistically, trained monocytes show reduced expression of adhesion molecules (ICAM-1) and chemoattractants (CCL2) due to the dampening of TLR signaling adapters, along with the restoration of cellular housekeeping functions like pexophagy​. These trained monocytes also upregulate the surface protein CD24, which can engage Siglec-10 on other immune cells to transmit inhibitory signals. Through this mechanism, CD24^hi^-trained monocytes curb the activity of neutrophils, T cells, and B cells in the plaque microenvironment, collectively reducing inflammation [98]. Although still in the preclinical phase, these cell-based therapies highlight the potential for modulating cell function within the plaque microenvironment.

## Conclusions

Our understanding of atherogenesis has evolved beyond a strictly lipid-centric view to encompass the intricate interplay of cellular and extracellular components within the plaque microenvironment. Advances in single-cell transcriptomics, fate-mapping models, and metabolomics have uncovered an unprecedented heterogeneity of cell phenotypes within atherosclerotic lesions, with some driving chronic inflammation while others resolution [56, 99, 100]. Translating these insights into new therapies will require the continued integration of cutting-edge technologies. Multi-omics approaches provide a comprehensive molecular landscape of atherosclerosis, while high-resolution imaging techniques, such as MALDI mass-spectrometry imaging, enable spatial mapping of key metabolic signatures. Furthermore, artificial intelligence and systems biology are becoming indispensable tools for deciphering the complex regulatory networks that govern atherosclerosis progression. These emerging insights could guide the development of precision therapies, such as those that enhance efferocytosis and promote collagen assembly while suppressing pathways that contribute to plaque expansion and rupture. Ultimately, atherosclerotic plaques are highly dynamic microenvironments where continuous interactions between immune cells, vSMCs, and ECM components shape disease outcomes. Targeting the plaque microenvironment through modulation of cell phenotypes, matrix remodeling, or precision drug delivery represents a promising strategy to mitigate plaque instability and prevent acute cardiovascular events.

Panel A: Endothelial cell activation augments the expression of endothelial adhesion molecules that bind cognate molecules/receptors on the leukocytes, resulting in tethering, rolling, adhesion, and diapedesis toward the subendothelial space.

Panel B: Stable Plaque: Efferocytosis by macrophages release pro-resolving mediators such as TGFβ and IL10 that promote vSMC quiescence. The presence of a basement membrane including collagen IV and laminin mitigate inflammation via integrin α2β1 and α6β1. The accumulation of non-smooth muscle fibroblast-like cells within the fibrous cap has recently been found in a stable plaque microenvironment.

Panel C: Unstable Plaque: Defective efferocytosis and an inflamed microenvironment favors plaque instability. The inefficient removal of apoptotic cells and activation of macrophages by oxLDL triggers the release of the proinflammatory mediators TNFα and IL-1β, which promotes the synthetic phenotype in vSMCs. The abnormal deposition of transitional ECM proteins such as fibronectin and vitronectin augment the inflammatory response in vSMCs and endothelial cells mediated by integrins α5β1 and αvβ3 signaling. The MMPs released by dedifferentiated vSMCs promotes ECM degradation and thins the fibrous cap.

Panel D: Atherosclerosis Initiation and Progression: At the initial stage of the atherosclerosis, the persistent retention of LDL, followed by its oxidation, activates endothelial cells and drives leukocyte recruitment. Along with the appearance of foam cells, vSMCs migrate and proliferate towards the intimal layer, expanding lesion size. However, significant death of macrophages, defective efferocytosis of remaining macrophages, vSMC phenotypic switching, and ECM remodeling at the atherosclerotic microenvironment drive the thinning of the fibrous cap, making plaque unstable and prone to rupture.

## Key References

- Rohwedder I, Montanez E, Beckmann K, Bengtsson E, Dunér P, Nilsson J, et al. Plasma fibronectin deficiency impedes atherosclerosis progression and fibrous cap formation. EMBO molecular medicine. 2012;4(7):564 − 76.
-
  - This study demonstrates that fibronectin has a dual role in atherosclerosis: initially, it drives atherosclerotic lesion formation, but also drives plaque stabilization by augmenting the recruitment of smooth vascular muscle cells.
- Ridker PM, Everett BM, Thuren T, MacFadyen JG, Chang WH, Ballantyne C, et al. Antiinflammatory therapy with canakinumab for atherosclerotic disease. New England journal of medicine. 2017;377(12):1119-31.
-
  - This is a randomized, double-blind trial that tested the inflammatory hypothesis in atherosclerosis using a therapeutic monoclonal antibody targeting interleukin-1β. It provided invaluable evidence on antiinflammatory therapies as a potential treatment strategy to lower this risk of cardiovascular disease, independent of lipid-level lowering.
- Fidler TP, Dunbar A, Kim E, Hardaway B, Pauli J, Xue C, et al. Suppression of IL-1β promotes beneficial accumulation of fibroblast-like cells in atherosclerotic plaques in clonal hematopoiesis. Nature cardiovascular research. 2024;3(1):60–75.
-
  - This study provides a potential mechanistic insight into the beneficial action of anti-inflammatory therapy, interleukin-1β inhibition with canakinumab, as seen in the CANTOS trial. The study showed IL-1β-mediated crosstalk between myeloid cell and stromal cells including endothelial, fibroblast, and smooth muscle cells in the setting of clonal hematopoiesis of indeterminate potential. Interestingly, researchers discovered the aggregation of non-smooth muscle fibroblast-like cells at the fibrous cap to stabilize the plaque following anti-IL-1β.

## Data Availability

No datasets were generated or analysed during the current study.
